# Granger-Causality Inference of the Existence of Unobserved Important Components in Network Analysis

**DOI:** 10.3390/e23080994

**Published:** 2021-07-30

**Authors:** Heba Elsegai

**Affiliations:** Department of Applied Statistics, Faculty of Commerce, Mansoura University, Mansoura City 35516, Egypt; dr.heba.elsegai@mans.edu.eg

**Keywords:** Granger-causality, rPDC, DPC, degree centrality, unobserved important nodes, network inference

## Abstract

Detecting causal interrelationships in multivariate systems, in terms of the Granger-causality concept, is of major interest for applications in many fields. Analyzing all the relevant components of a system is almost impossible, which contrasts with the concept of Granger causality. Not observing some components might, in turn, lead to misleading results, particularly if the missing components are the most influential and important in the system under investigation. In networks, the importance of a node depends on the number of nodes connected to this node. The degree of centrality is the most commonly used measure to identify important nodes in networks. There are two kinds of degree centrality, which are in-degree and out-degree. This manuscrpt is concerned with finding the highest out-degree among nodes to identify the most influential nodes. Inferring the existence of unobserved important components is critical in many multivariate interacting systems. The implications of such a situation are discussed in the Granger-causality framework. To this end, two of the most recent Granger-causality techniques, renormalized partial directed coherence and directed partial correlation, were employed. They were then compared in terms of their performance according to the extent to which they can infer the existence of unobserved important components. Sub-network analysis was conducted to aid these two techniques in inferring the existence of unobserved important components, which is evidenced in the results. By comparing the results of the two conducted techniques, it can be asserted that renormalized partial coherence outperforms directed partial correlation in the inference of existing unobserved important components that have not been included in the analysis. This measure of Granger causality and sub-network analysis emphasizes their ubiquitous successful applicability in such cases of the existence of hidden unobserved important components.

## 1. Introduction

Several statistical analysis techniques have been developed to detect interrelationships in multivariate systems. Examples of such techniques are based on mutual information [1,2,3,4,5], autoregressive processes [6,7,8], coherence [9,10,11], and recurrence in state space [12,13,14]. On the other hand, for causal inference, the concept of Granger causality originated from econometrics [15,16,17,18,19]. The most well-known frequency and time-domain techniques based on this concept are renormalized partial directed coherence (rPDC) [20] and directed partial correlation (DPC) [21], respectively.

Investigation of causal interactions between multiple processes is especially relevant in financial markets, neurosciences, and other different areas of application. The investigation of interrelationships among processes is of particular interest, that is, understanding the underlying interaction network structure promises to uncover the basic mechanisms of the underlying system. In this study, Granger causality [16,17,18] is used to investigate causal interactions. The concept of Granger causality is probabilistic, as it is formulated in terms of the idea of predictability. More precisely, this concept relies on the intuitive notion that causes always precede their effects in time.

Notably, Granger (1969) introduced this causality concept claiming that it relies on observing all relevant processes, i.e., the information contained in the entire universe. This, however, cannot be in practice achieved. As a consequence, not observing some components might, in turn, lead to misleading results, particularly if the missing components are the most influential and important in the system under investigation. The main problem appears when such important nodes are not included in the analysis under investigation, or if they are not even observable.

In networks, the importance of a node depends on the number of nodes that are connected to this node [22]. The degree of centrality is the most commonly used measure to identify important nodes in networks [23,24,25,26,27,28,29]. In directed networks, a single degree metric is divided into two metrics, which are in-degree and out-degree [27]. The in-degree metric is the measure of the number of edges that point towards the node of interest. The out-degree metric is the measure of the number of links that the node of interest points towards. This manuscript is concerned with finding the highest out-degree among nodes to identify the most influential nodes. Inferring the existence of unobserved important components is critical in many multivariate interacting systems. To this aim, two recent causality techniques were developed based on the concept of Granger causality. The first one is called partial directed coherence (PDC), which was developed by Baccalla and Sameshima (2001) as a time-domain causal measure [30]. On the other hand, a frequency-domain causal technique known as renormalized partial directed coherence (rPDC) allows for superior normalization and statistics [20]. These techniques were compared in different scenarios. This was done to investigate which of these techniques could accurately observe the existence of unobserved important components. This manuscript also discusses the implications of not observing important components on the resulting causal network.

This manuscript is structured as follows. The comparison methods, rPDC and DPC, are introduced in the first section. The results of simulations with the implications of the Granger-causality techniques are discussed in the second section. The sensitivity analysis of the results is presented in the third section, followed by a conclusion.

## 2. Comparison Methods

Through this section, the comparison methods employed in this manuscript are summarized. The concept of Granger-causality together with vector autoregressive (VAR) processes are presented in the first sub-section. The time-domain causality technique, Directed Partial Correlation (DPC), is introduced in the second sub-section of this section. In the third part, the frequency-domain causal technique renormalized Partial directed coherence (rPDC) is presented.

### 2.1. Vector Autoregressive Model (VAR) and Granger-Causality

The concept of Granger-causality is based on two main aspects. The first is the idea that “causes precede their effects in time”, which is known as temporal precedence. The second is that the information set included in the entire universe must be taken into consideration in the analysis.

The Granger-causality concept is assessed based on the class of vector autoregressive models. Such models describe linear relations between processes [16]. A process $X_{j}$ is considered as a Granger-causal for process $X_{i}$ if the prediction of the latter can be improved by gaining past knowledge of the first process $X_{j}$. The improvement, here, refers to a smaller variance of forecasting error [21]. The vector autoregressive models are classified, typically, according to graphical models class [31,32]. Graphical models provide a common tool used for visualizing as well as analysing connectivity patterns among multivariate processes with respect to graphs [10,31,32,33,34,35]. A graph is a way of visualizing a pair of set of vertices and set of edges. A graph is defined, mathematically, as G=(V,E), where *V* refers to Vertices and *E* refers to edges. The vertices correspond to the graph nodes, which represent the multivariate system components. Graphically, two vertices are considered to be connected, that is, a link exists between the two, with this link then known as an edge. In terms of directionality, an edge can be directed, that is, a direct interaction is detected between the corresponding nodes, otherwise it is undirected. The graph could be weighted if there are numbers assigned to edges, otherwise it is not. The assigned weights on the edges reflect the interaction strength between nodes.

The *n*-dimensional vector autoregressive process of order *p*, denoted by VAR[*p*], is given by

(1) $$X\left(t\right)=\sum_{r=1}^{p}\;A\left(r\right)\;X\left(t-r\right)+\varepsilon \left(t\right),$$

with n×n coefficient matrices A(r),r=1,…,p . The *n*-dimensional independent Gaussian white noise is denoted by ε(t), where the covariance matrix Σ is non-singular and ε(t)∼N(0,Σ). It is well-known that the class of a VAR process is stationary, that is, the roots of the lag polynomial are found outside the unit circle, for details and examples refer to [36].

### 2.2. Directed Partial Correlation (DPC): A Granger-Causal Time-Domain Technique

To provide a Granger-causal measure in the time-domain, directed partial correlation (DPC) was developed by Eichler (2005) [21]. This technique can be used, effectively, to measure the strength of causal effects among multiple components [21].

The inference of causal interactions from time-series data needs fitting VAR[*p*] models to be fitted based on the least-squares estimation method [21], which is utilized throughout this manuscript. For observations $X_{V}\left(1\right),\cdots ,X_{V}\left(T\right)$ from a *d*-dimensional multiple time series $X_{V}$, let ${\hat{R}}_{p}={\left({\hat{R}}_{p}\left(h,\nu \right)\right)}_{h,\nu =1,\cdots ,p}$ be the pd × pd matrix composed by sub-matrices [21]

(2) $${\hat{R}}_{p}\left(h,\nu \right)=\frac{1}{T-p}\;\sum_{t=p+1}^{T}\;X\left(t-h\right)\;X{\left(t-\nu \right)}^{T},$$

where *T* refers to the number of observations and h,ν=1,⋯,p. Similarly, ${\hat{r}}_{p}$ is set to be such that ${\hat{r}}_{p}=\left({\hat{R}}_{p}\left(0,1\right),\cdots ,{\hat{R}}_{p}\left(0,p\right)\right)$. After that, the least-squares estimates of the autoregressive coefficients are given by

(3) $${\hat{A}}_{ij}\left(h\right)=\sum_{\nu =1}^{p}\;{\left({\hat{R}}_{p}\right)}^{-1}\left(h,\nu \right)\;{\hat{r}}_{p}\left(\nu \right),$$

where h=1,⋯,p, while the covariance matrix Σ of the error ϵ(t), which refers to the *n*-dimensional independent Gaussian white noise regarding VAR[*p*] model, is estimated by

(4) $$\hat{\Sigma }=\frac{1}{T}\;\sum_{t=p+1}^{T}\;\hat{\varepsilon }\left(t\right)\;\hat{\varepsilon }{\left(t\right)}^{T},$$

where

(5) $$\hat{\varepsilon }\left(t\right)=X\left(t\right)-\sum_{h=1}^{p}\;A\left(h\right)\;X\left(t-h\right)$$

are the least-squares residuals. Note that the coefficients $A_{ij}\left(h\right)$ depend on the unit of measurement of $X_{i}$ and $X_{j}$. This, in turn, makes the comparisons, in terms of the strength of causal interactions among processes, unsuitable [21]. To this end, Eichler (2005) [21] developed the DPC technique as a tool to measure the causal interactions’ strength. The DPC $\pi_{ij}\left(h\right)$, for h>0, is defined as the correlation between $X_{i}\left(t\right)$ and $X_{j}\left(t-h\right)$, after the linear effects of all other variables included in the vector process $X_{V}$ are removed. On the other hand, $\pi_{ij}\left(h\right)=\pi_{ij}\left(-h\right)$ for h<0. Furthermore, it has been shown in [21] that estimates for the DPCs $\pi_{ij}\left(h\right)$, where h>0, can be obtained from the parameter estimates of a VAR[*p*] model. This was done by re-scaling the coefficients $A_{ij}\left(h\right)$

(6) $${\hat{\pi }}_{ij}\left(h\right)=\frac{{\hat{A}}_{ij}\left(h\right)}{\sqrt{{\hat{\Sigma }}_{ii}\;{\hat{\rho }}_{jj}\left(h\right)}}\;\;\mathrm{for}\;\;j\to i\;,$$

where

(7) $${\hat{\rho }}_{jj}\left(h\right)={\hat{K}}_{jj}+\sum_{\nu =1}^{h-1}\;\sum_{k,l\in V}\;{\hat{A}}_{kj}\left(\nu \right)\;{\hat{K}}_{kl}\;{\hat{A}}_{lj}\left(\nu \right)+\frac{{\hat{A}}_{ij}{\left(h\right)}^{2}}{{\hat{\Sigma }}_{ii}}.$$

The matrix $\hat{K}={\hat{\Sigma }}^{-1}$, where ${\hat{\Sigma }}^{-1}$ denotes the estimated covariance matrix $\hat{\Sigma }$ inverse with respect to the residual noise processes.

To decide whether an estimated DPC value is significant, the researcher conducted a statistical evaluation scheme based on the idea of bootstrapping, with the confidence interval constructed as follows:

1. Generate a number of bootstrap surrogates *B* of a length that is similar to a practical data set. Roughly, 1000 bootstrap surrogates, as a minimum, is usually enough for accurate computation of confidence intervals, as proposed by Efron and Tibshirani [37]. Throughout this manuscript, *B* is set to 10,000 bootstrap surrogates. The surrogates are generated using the well-known non-parametric method, Amplitude Adjusted Fourier Transform (AAFT) [38,39]. The AAFT method works based on generating data from a Gaussian, stationary, and linear stochastic process [40]. Generating *B* surrogates is done based on the following algorithm [40,41]:
  (a) Re-scaling the data according to normal distribution. This re-scaling is done by generating the time series according to Gaussian distribution. This is based on a simple rank ordering, which is then arranged with respect to the order of data.
  (b) Constructing a Fourier transformed surrogate for this re-scaled data.
  (c) Re-scaling the final obtained surrogate in terms of the data distribution. The data is then arranged in terms of the rank of the Fourier transformed surrogate.
  The advantage of using this algorithm is that it approximately conserves the distribution as well as the power spectrum of the data [40,41]. The AAFT method is implemented using the Tisean package found in http://www.mpipks-dresden.mpg.de/tisean/ (accessed on 15 February 2020) [39]. Note that the above-mentioned algorithm is employed iteratively based on the Tisean package until no more improvement could be made [39].
2. Estimating the DPC value for each *B* bootstrap surrogate to obtain a bootstrap sampling distribution, i.e., ${\left\{{\hat{\tau }}_{r}^{⋆}\right\}}_{r=1,\cdots ,B}$. To achieve the (1−α)100 percentile bootstrap confidence interval for $\hat{\tau }$, the values of sampling distribution, ${\hat{\tau }}_{r}^{⋆}$, are arranged in ascending order. After that, the points of α as well as (1−α) percentages are chosen to be the end points of the confidence interval, to yield [${\hat{\tau }}_{r}^{⋆}\left(\alpha B\right),{\hat{\tau }}_{r}^{⋆}\left(\left(1-\alpha \right)B\right)$] [42]. Approximately, the resulting 95% confidence interval for B=10000 is [${\hat{\tau }}^{⋆}\left(500\right),{\hat{\tau }}^{⋆}\left(9500\right)$].
3. Finally, if the estimated DPC value is found to be outside the confidence interval, it means that the estimated DPC value is significant and different from zero.

### 2.3. Renormalized Partial Directed Coherence (rPDC): A Granger-Causal Frequency- Domain Technique

The well-known Granger-causal technique that was introduced in the frequency domain is called Partial directed coherence (PDC) [43]. This technique is based on VAR modelling of the signals employing appropriate VAR model order *p* [43]. Some drawbacks have been detected when performing PDC analysis, as outlined in Schelter et al. (2009) [20]. Therefore, a renormalized version of PDC has been developed as a means to detect not only Granger-causal interactions but also the strengths of the directed causal interactions among components in a network [20].

In order to measure causal interactions in terms of Granger-causality in frequency-domain, the Fourier transform

(8) $$A\left(\omega \right)=I-\sum_{r=1}^{p}\;a\left(r\right)\;e^{-i\omega r},$$

of the coefficients a(r) of Equation (1) [20,33] is performed. To introduce rPDC consider the two-dimensional vector

(9) Zkj(ω)=Re(Akj(ω))Im(Akj(ω)),

which consists of two main parts, real and imaginary, of the Fourier transformed coefficients. The corresponding estimator would be ${\hat{Z}}_{kj}\left(\omega \right)$, with ${\hat{A}}_{kj}\left(\omega \right)$ replacing $A_{kj}\left(\omega \right)$, Gaussian distributed with the mean $Z_{kj}\left(\omega \right)$ and the following covariance matrix

(10) $$\begin{array}{c} V_{kj}\left(\omega \right)/N=\sum_{l,m=1}^{p}\;R_{jj}^{-1}\left(l,m\right)\;\Sigma_{kk}\times \frac{1}{N}\left[\begin{array}{cc} \cos(l\omega )\;\cos(m\omega ) & \cos(l\omega )\;\sin(m\omega )\\ \sin(l\omega )\;\cos(m\omega ) & \sin(l\omega )\;\sin(m\omega ) \end{array}\right], \end{array}$$

where *N* denotes the number of data points and R denotes the covariance matrix of the VAR process. Then, the renormalized partial directed coherence is defined by

(11) $$\lambda_{kj}\left(\omega \right)=Z_{kj}{\left(\omega \right)}^{\prime }\;V_{kj}^{-1}\left(\omega \right)\;Z_{kj}\left(\omega \right).$$

If $\lambda_{kj}\left(\omega \right)=0$, then a Granger-causal influence of process $x_{j}$ on process $x_{k}$ taking into account all other processes, i.e., $\left\{x_{l},l\neq j,k\right\}$, would be rejected at frequency ω. The critical value for a α-significance level for $\lambda_{kj}\left(\omega \right)=0$ is given by $\chi_{2,1-\alpha }^{2}/N$ [20], where the quantile 1−α of the $\chi^{2}$-distribution with two degrees of freedom is denoted by $\chi_{2,1-\alpha }^{2}$.

## 3. Simulations

**Figure 1:**
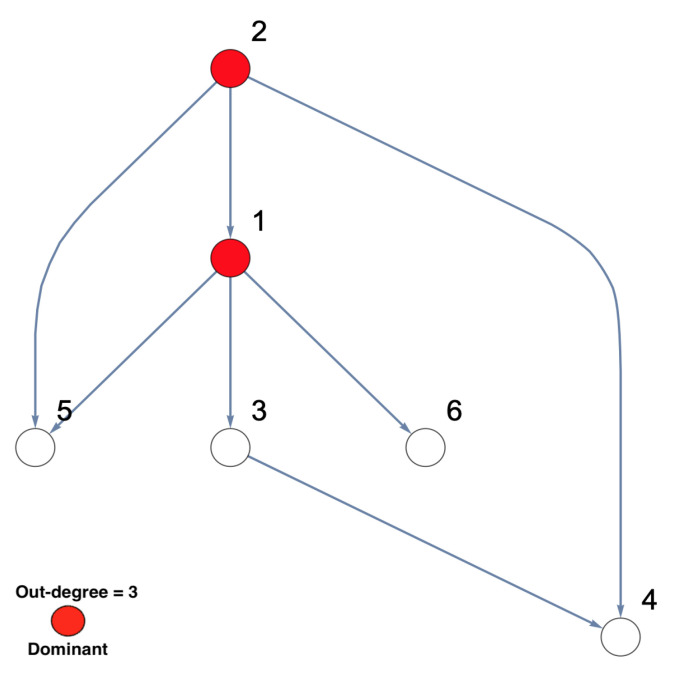
The simulated network structure of the underlying investigated system. This six-dimensional network structure shows that Nodes 1 and 2 are important nodes according to their out-degrees. In other words, these two nodes influence all other nodes in the underlying investigated network, either directly or indirectly.

This study aims to investigate the implications of the existence of unobserved important components, which have not been included in the analysis, on the inferred network structure, as well as to what extent the existence of unobserved important components can be accurately inferred. For a deep understanding of the dependence structure in the presence of unobserved important components, sub-network analysis has been suggested [44]. The simulated network structure that represents the underlying investigated system is presented in Figure 1. In the context of this manuscript, the important components are defined in terms of the highest number of out-degrees of a node. In Figure 1, it can be seen that the causality structure of the underlying system shows that Nodes 1 and 2 are considered important nodes according to the number of their out-degrees, i.e., out-degree = 3. More precisely, Nodes 1 and 2 not only directly influence most other nodes but also indirectly influence the rest of the network. The underlying structure can be considered a tree structure in the form of layers, which is similar to the structure of chain networks.

In the following, the results of employing both rPDC and DPC Granger-causality techniques are presented, such that four scenarios are considered in the analysis. These scenarios are as follows: not observing Node 1, not observing Node 2, not observing Nodes 1 and 2, and not observing Nodes 1, 2, and 3. Note that nodes were omitted systematically in sub-network analysis.

### 3.1. Results: rPDC Granger-Causality Technique

**Figure 2:**
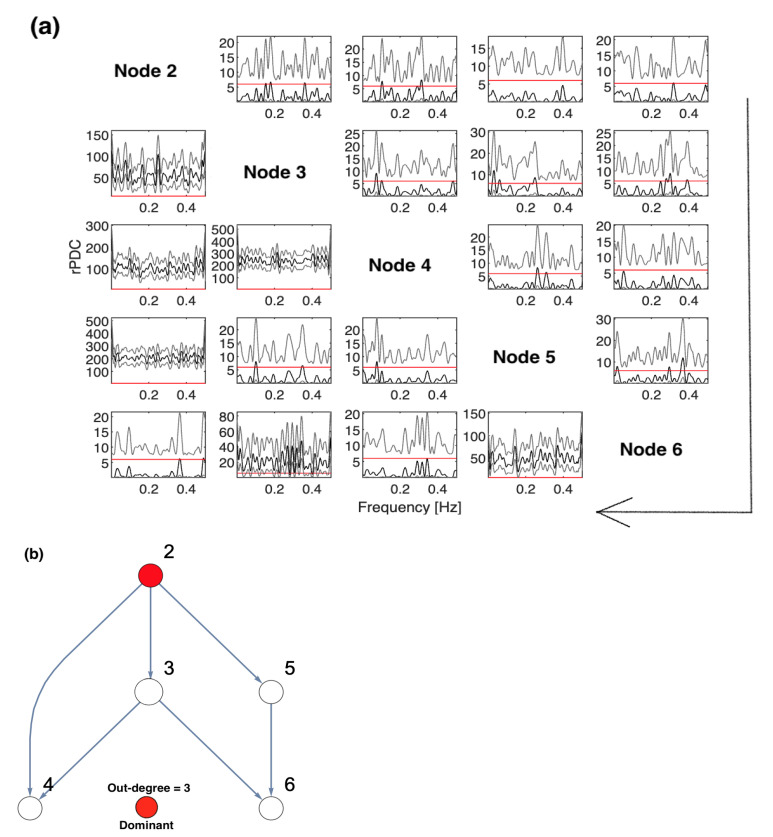
The result of the rPDC technique after excluding Node 1 from the analysis: (**a**) presents the results of rPDC in a matrix form, while (**b**) presents the results as a causal network. In (**a**), the diagonal represents the node number with respect to each column and row, while the large arrow drawn around the figure shows the direction of the influence of each node on the other nodes, vertically. In the sub-figures, the *x*-axis corresponds to the frequency, while the *y*-axis corresponds to the calculated rPDC value. It can be noticed that four line realizations appear in the sub-figures. The black line realization represents rPDC values, while the gray regions refer to the corresponding 95% confidence intervals of a single realization. Importantly, the red line marks the critical value at a 5% significant level. Note that the node color in red corresponds to the important node, as the highest number of out-degrees in the network is for Node 2.

The results of conducting rPDC based on sub-network analysis, such that Node 1 is omitted, are displayed in Figure 2, where two different representations are shown. Figure 2a presents the results of rPDC in a matrix form, while Figure 2b presents the results as a causal network. In Figure 2a, the diagonal represents the node number with respect to each column and row, while the large arrow drawn around the figure shows the direction of the influence of each node on other nodes, vertically. In the sub-figures, the *x*-axis corresponds to the frequency, while the *y*-axis corresponds to the calculated rPDC value. It can be noticed that four line realizations might appear in some of the sub-figures. The black line realization represents the rPDC values, while the gray regions refer to the corresponding 95% confidence intervals of a single realization. Importantly, the red line marks the critical value at the 5% significant level. Note that the node colour in red corresponds to the important node, as the highest number of out-degrees in the network is for Node 2. It is noteworthy that the rPDC values are not normalized, so the values are large, but this does not affect the results. The main interest in this manuscript is the significant detection of causal interaction, whether there is an influence or not. This is in the case of not observing components that are important in the underlying simulated system. Furthermore, a rPDC value is considered to be significant if, and only if, the black line realization is completely above the red line for all different frequencies. Note that the red line sometimes can be exactly on the *x*-axis so that it cannot be clearly seen.

On the other hand, to deeply understand the results, they are represented as a network with interacting nodes in Figure 2b. The important nodes, highlighted in red, are determined according to their out-degrees, i.e., out-degree = 3. Node 2 has three outgoing directed links, while Node 3 has two outgoing directed links. Other nodes have only one outgoing directed link or nothing. Therefore, the important node of the observed five-dimensional network is Node 2. It can be observed that Node 2 influences Nodes 3, 4, and 5 directly and influences Node 6 indirectly. It can be seen that Node 3 influences Node 6 on behalf of the unobserved Node 1, which is because Node 1 directly influenced both Nodes 5 and 6, as shown in Figure 1. All other links presented in the underlying simulated system, see Figure 1, are significantly detected in the inferred five-dimensional sub-system, see Figure 2.

**Figure 3:**
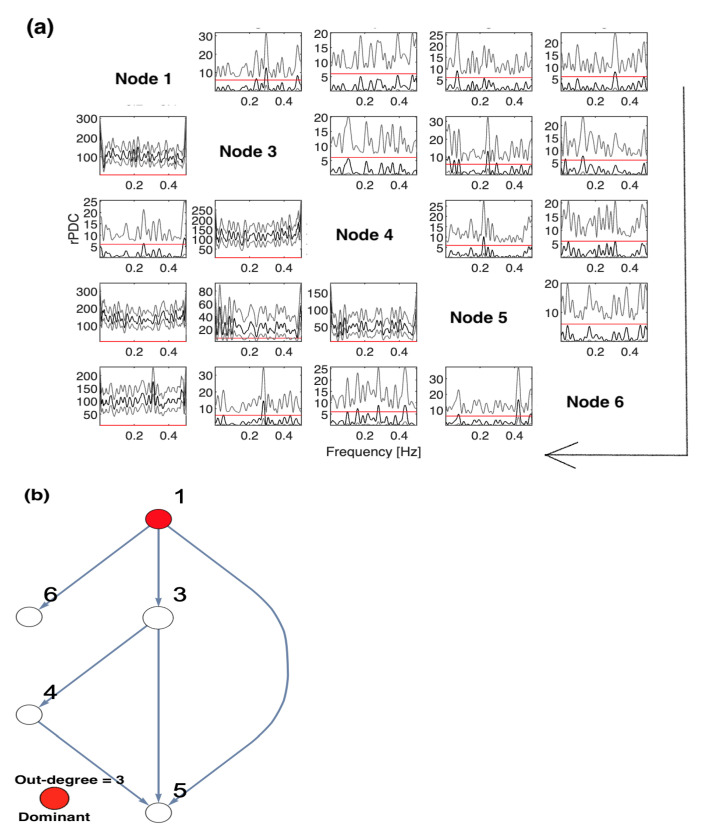
The figure presents the results of conducting rPDC analysis for the observed five-dimensional network after Node 2 is omitted: (**a**) presents the results of rPDC in a matrix form, while (**b**) presents the results as a causal network. In (**a**), the diagonal represents the node number with respect to each column and row, while the large arrow drawn around the figure shows the direction of the influence of each node on the other nodes, vertically. In the sub-figures, the *x*-axis corresponds to the frequency, while the *y*-axis corresponds to the calculated rPDC value. It can be noticed that four line realizations might appear in some sub-figures. The black line realization represents rPDC values, while the gray regions refer to the corresponding 95% confidence intervals of a single realization. Importantly, the red line marks the critical value at a 5% significant level. Note that the node color in red corresponds to the important node, as the highest number of out-degrees in the network is for Node 2.

Figure 3 presents the results of rPDC with its corresponding graphical representation of the implications of not observing Node 2 only, while all other nodes are included in the analysis. The resulting five-dimensional sub-system shows that all links are present as in the underlying system, except the link 4→5. This causal link exists because Node 2 influenced Nodes 4 and 5 directly and via Node 3 indirectly. Therefore, the effect of Node 3 on Node 4 leads to the influence of Node 4 on Node 5.

**Figure 4:**
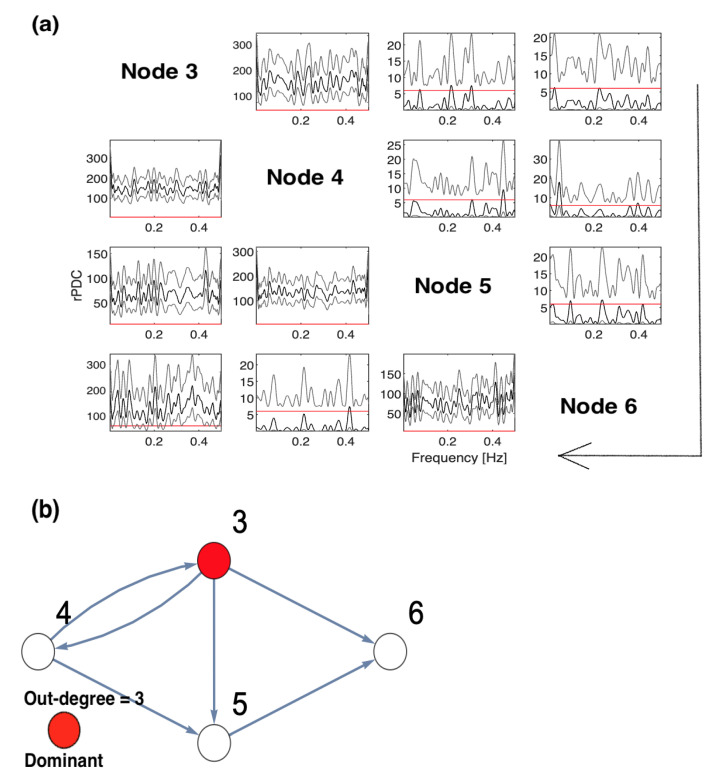
The figure presents the results of conducting rPDC analysis for the observed four-dimensional network after Nodes 1 and 2 are omitted: (**a**) presents the results of rPDC in a matrix form, while (**b**) presents the results as a causal network. In (**a**), the diagonal shows the node number with respect to each column and row, while the large arrow drawn around the figure shows the direction of the influence of each node on other nodes, vertically. In the sub-figures, the *x*-axis corresponds to the frequency, while the *y*-axis corresponds to the calculated rPDC value. It can be noticed that four line realizations might appear in some sub-figures. The black line realization represents rPDC values, while the gray regions refer to the corresponding 95% confidence intervals of a single realization. Importantly, the red line marks the critical value at a 5% significant level. Note that the node color in red corresponds to the important node, as the highest out-degree in the network is for Node 3. Furthermore, the results show the feedback causal pattern between Nodes 3 and 4.

**Figure 5:**
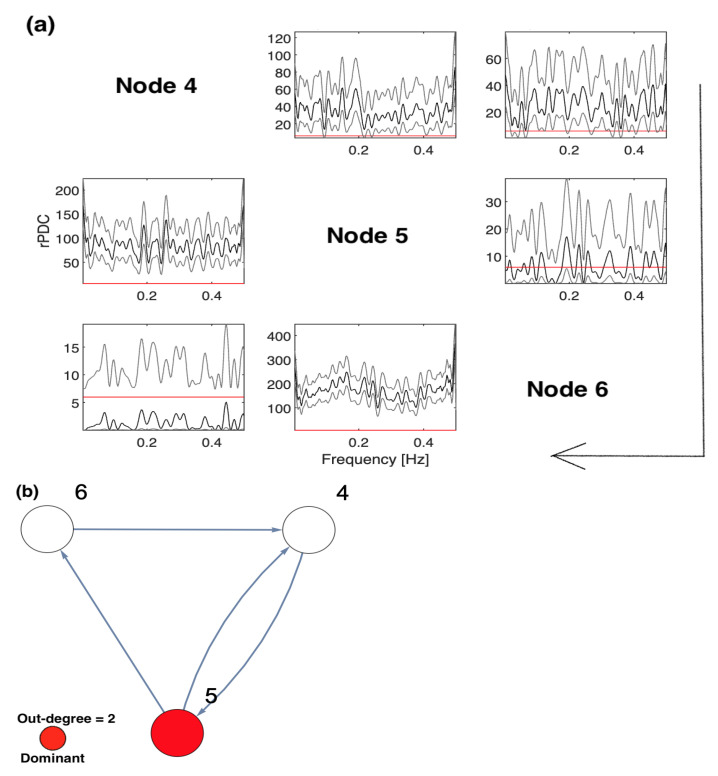
The figure presents the results of conducting rPDC analysis for the observed three-dimensional network after Node 3 is omitted: (**a**) presents the results of rPDC in a matrix form, while (**b**) presents the results as a causal network. In (**a**), the diagonal shows the node number with respect to each column and row, while the large arrow drawn around the figure shows the direction of the influence of each node on other nodes, vertically. In the sub-figures, the *x*-axis corresponds to the frequency, while the *y*-axis corresponds to the calculated rPDC value. It can be noticed that four line realizations might appear in some sub-figures. The black line realization represents rPDC values, while the gray regions refer to the corresponding 95% confidence intervals of a single realization. Importantly, the red line marks the critical value at a 5% significant level. Note that the node color in red corresponds to the important node, as the highest out-degree in the network is for Node 5. Furthermore, the results show the feedback causal pattern between Nodes 4 and 5.

The implications of omitting the important Nodes 1 and 2 are presented in Figure 4. These are the results of rPDC with its corresponding graphical representation of the four-dimensional sub-system. It can be observed that Node 3 influences each of Nodes 4, 5, and 6. Interestingly, there is a feedback interaction structure that appears in the four-dimensional sub-system between Nodes 3 and 4. The results, additionally, show that Node 3 becomes the important component in the network. Therefore, the rPDC analysis was conducted for the three-dimensional sub-system after Node 3 is omitted. The results are presented in Figure 5. It can be observed that there is a causal feedback structure between Nodes 4 and 5.

In fact, combining the results presented in Figure 4 and Figure 5 for the four-dimensional and three-dimensional sub-systems, the following can be concluded. According to these figures, the first feedback structure is between Nodes 3 and 4, and the second is between Nodes 4 and 5. This provides, firstly, an indication that there have been unobserved important components that were not included in the analysis. Secondly, the important unobserved components had a large influence on Nodes 3, 4, and 5. This can be asserted by looking back into the original network, as Nodes 1 and 2 both influenced Nodes 3, 4, and 5, but Node 1 also influenced Node 6. Therefore, by taking the common nodes that have been influenced by both Nodes 1 and 2, the accurate underlying causal network structure can be inferred.

### 3.2. Results: DPC Granger-Causality Technique

**Figure 6:**
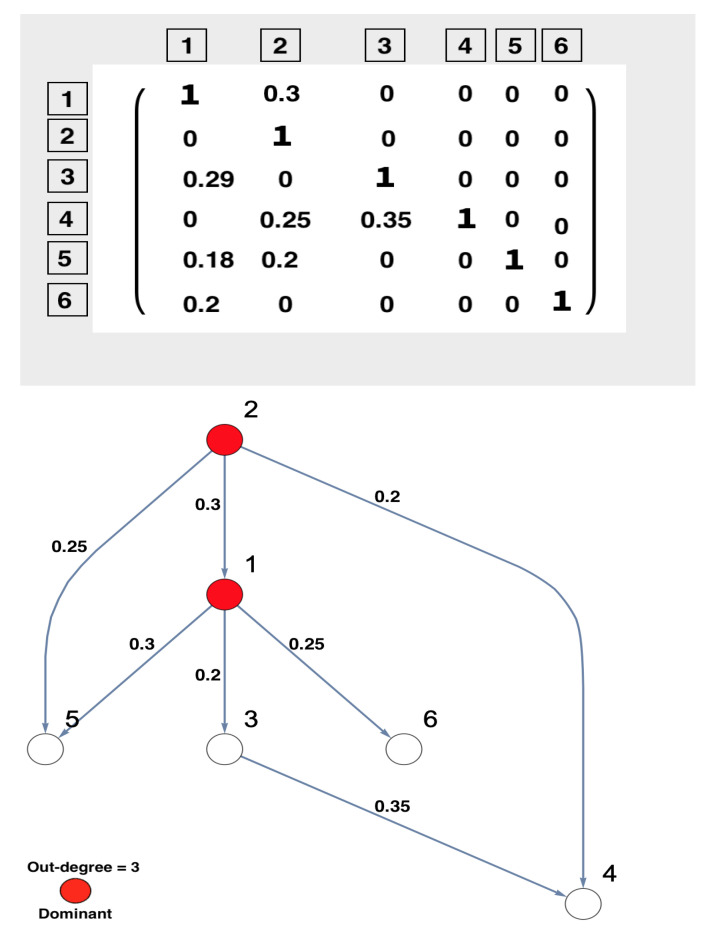
The figure presents the results of conducting DPC analysis for the underlying simulated six-dimensional system. The DPC matrix of the simulated underlying system and the corresponding graphical representation are presented. The numbers written in boxes correspond to the nodes’ number, whereas the simulated interaction coefficients in the DPC matrix are presented on the links of the network. This six-dimensional network structure shows that Nodes 1 and 2 are important nodes according to their out-degrees. In other words, these two nodes influence all the nodes in the network. The important nodes are highlighted in red according to their out-degrees.

**Figure 7:**
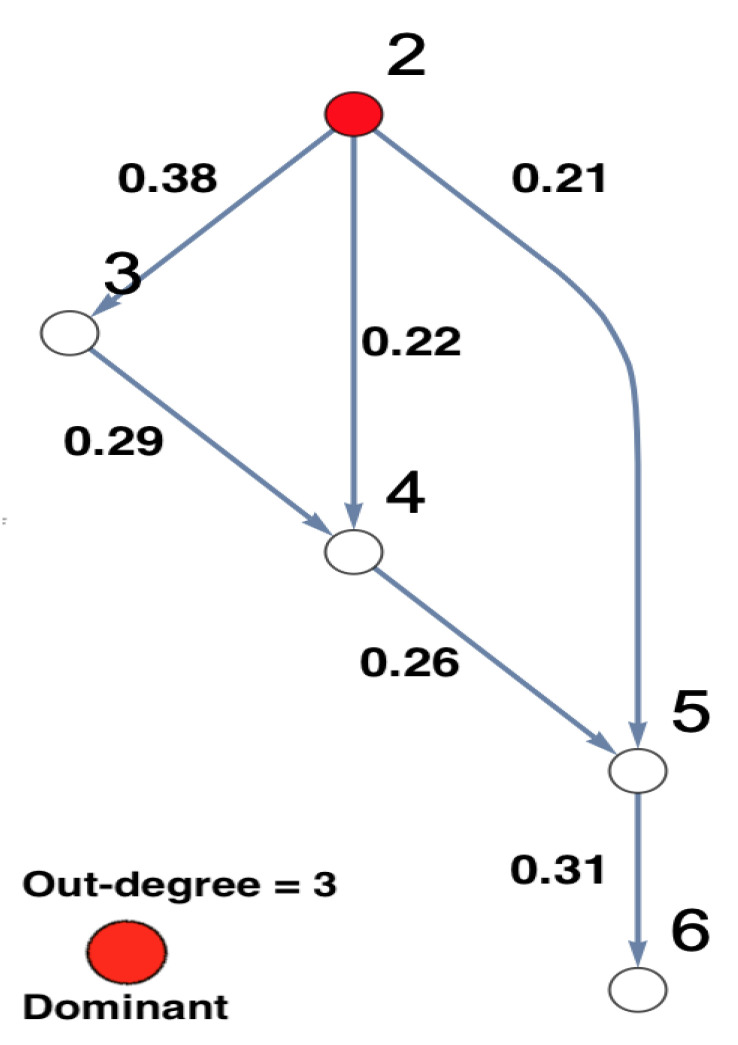
The figure presents the graphical representation of the results of conducting DPC analysis for the observed five-dimensional network after Node 1 is omitted. The observed interaction coefficients are presented on the links of the network. This five-dimensional network structure shows that Node 2 is important according to its out-degree, which is highlighted in red. It shows that Node 2 remains an important component in the network. The important node is highlighted in red.

The underlying simulated system is presented in a DPC matrix form. This is displayed in Figure 6, showing the interaction coefficients with its corresponding network structure. In this part, the same systematic analysis that was done based on the rPDC technique, see Section 11, is employed here based on DPC analysis. The systematic analysis starts with Node 1 being unobserved, see Figure 7. In the following, the reason for the presence of new causal links—2→3, 4→5, and 5→6—is illustrated. The link 2→3 is present because, in Figure 6, Node 2 is influencing Node 3 indirectly via Node 1. On the other hand, the link 4→5 is present because, in Figure 6, Node 2 is influencing Nodes 4 and 5 but is affecting Node 4 more strongly than Node 5, which forces Node 4 to influence Node 5. Furthermore, the link 5→6 is present because, in Figure 6, Node 2 is influencing Node 6 indirectly through Node 1. Node 2 influenced Node 5 directly and indirectly through Node 1, which in turn leads to the appearance of this link when Node 1 is omitted.

**Figure 8:**
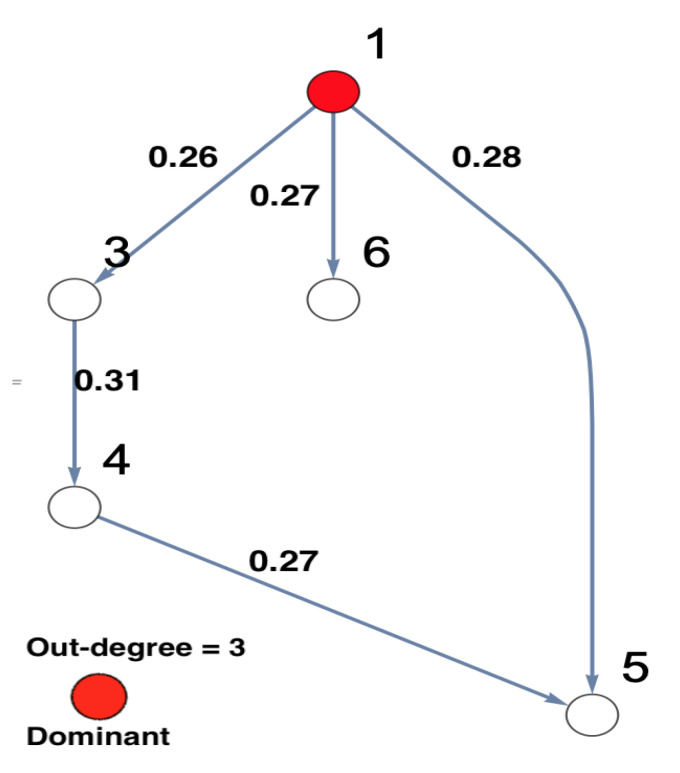
The figure presents the graphical representation of the results of conducting DPC analysis for the observed five-dimensional network after Node 2 is omitted. The observed interaction coefficients are presented on the links of the network. This five-dimensional network structure shows that Node 2 is important according to its out-degree, that is highlighted in red. It shows that Node 1 remains the important node in the network.

**Figure 9:**
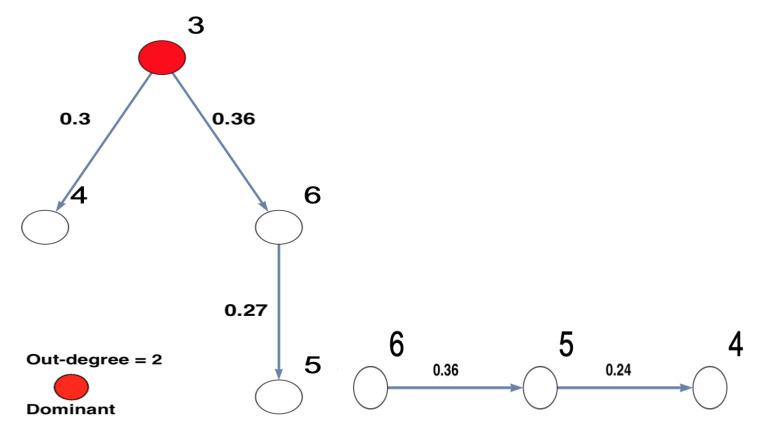
The figure presents the graphical representation of the results of conducting DPC analysis for the observed four-dimensional and three-dimensional sub-networks after Nodes 1, 2, and 3 are omitted. The observed interaction coefficients are presented on top of the links of the network. This five-dimensional network structure shows that Node 2 is being important according to its out-degree, that is highlighted in red.

On the other hand, the results of not observing Node 2 while all other nodes remain the same are shown in Figure 8. It can be seen that all links are present in Figure 8 as in Figure 6, except the link 4→5 is new. Furthermore, the results of both the four-dimensional sub-system—i.e., Nodes 1 and 2 are omitted—and the three-dimensional sub-system—i.e., Node 3 is omitted after omitting Nodes 1 and 2—were investigated. Figure 9 shows no clear evidence that there were unobserved important components. This is in contrast to the conclusion resulting from rPDC results, see Figure 4 and Figure 5. More precisely, the presence of a feedback interaction structure in the four-dimensional and three-dimensional sub-networks based on the rPDC technique, is considered potential evidence of the existence of unobserved important components. Furthermore, by having Node 6 link with other nodes in the sub-networks, an indication is given that Node 6 is also affected by the unobserved important components.

To sum up, these results show that rPDC outperforms DPC for inferring the existence of unobserved important components and for inferring the general true causal network structure of the underlying system. More precisely, the results of the DPC technique do not lead to any obvious conclusion that there is an unobserved important component. However, on the other hand, the rPDC technique reveals an indication of having unobserved important components that have not been taken into account in the analysis. Therefore, the true underlying causal structure can be potentially inferred, where important nodes are not included in the analysis.

## 4. Sensitivity Analysis

To demonstrate the validity of the inferred causal links, power and coverage analysis was conducted for each causal link in both directions between every two nodes. For this purpose, 100 realizations were simulated for each observed causal link. For the aim of testing for the significance of an estimated DPC value, the significance level of 5% was chosen so that a confidence interval of 95% was constructed for each combination in both directions.

For example, if x→y, then the null hypothesis of the statement “*x* does not influence *y*” is rejected, but it is true at a confidence of 95%. This is the case where the probability of obtaining a false positive link is at most 5%. The significance test is, similarly, employed for the other direction y→x, where the null hypothesis of the statement that “*y* does not influence *x*” is rejected, but it is false at a confidence of 95%. This case refers to true positives.

To validate the results, the validity is evaluated by power analysis. The power curve is drawn so that the ability to detect an accurate rejection of the null hypothesis is quantified. On the other hand, the fraction of false positives is controlled by coverage analysis [45].

**Figure 10:**
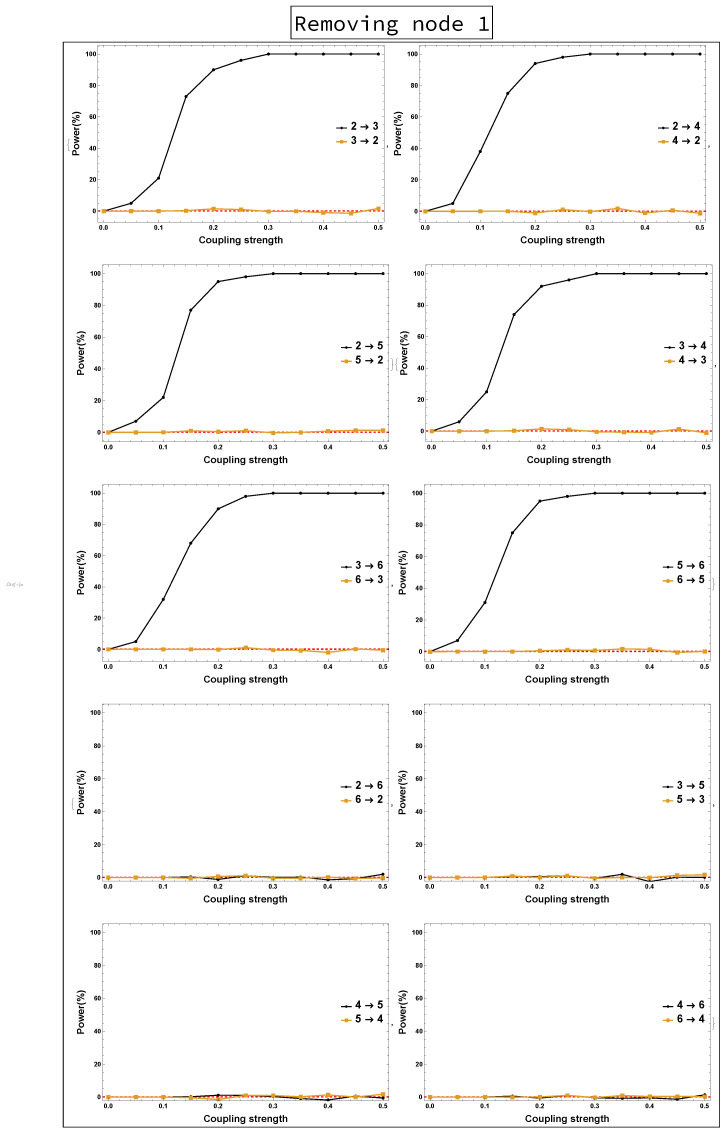
rPDC power curve. The figure presents three lines with different colors: dark-black line, light-orange line, and red-dashed line. The first two lines refer to the causal interactions in both directions, while the red-dashed line highlights 5% of the realizations. The *x*-axis presents the coupling strength, whereas the *y*-axis presents the percentage of realizations. The figure shows the results of power analysis based on rPDC analysis after Node 1 is omitted. The analysis is done for a relationship between any two nodes for both directions in the observed five-dimensional sub-network.

**Figure 11:**
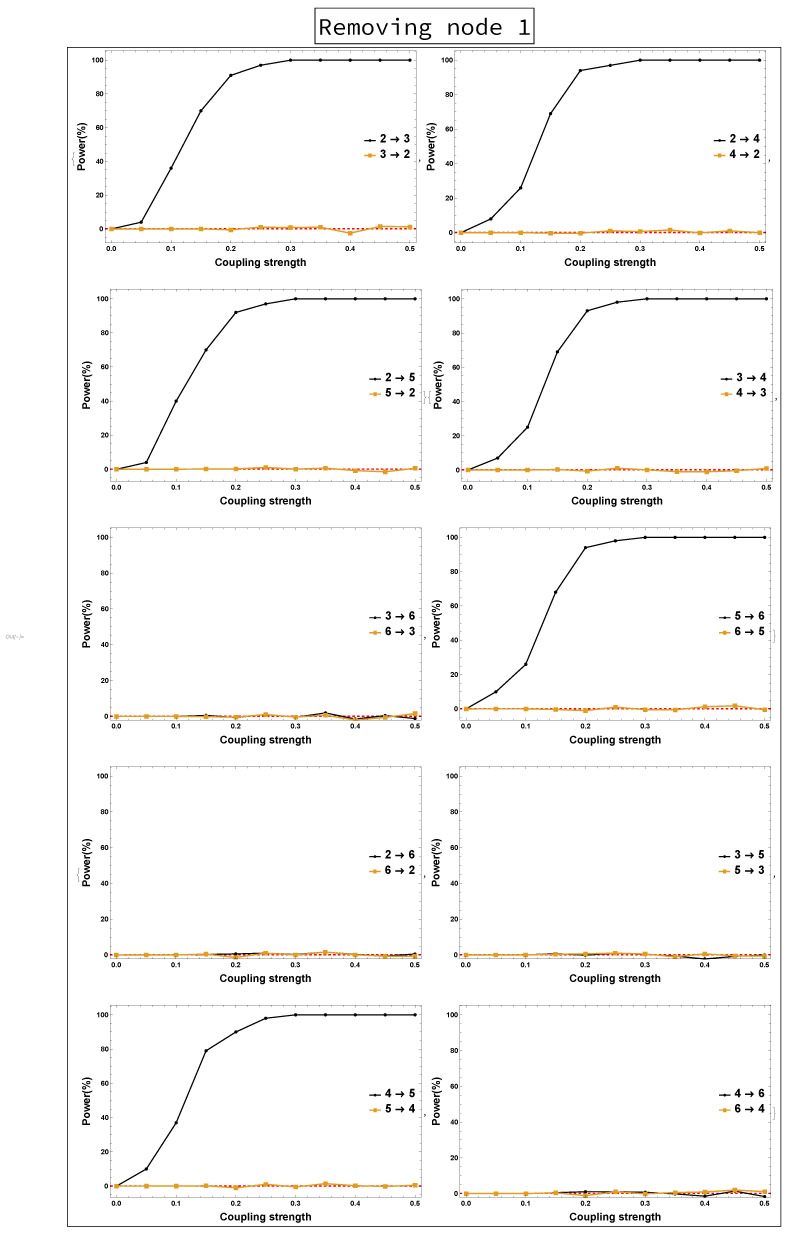
DPC power curve. The figure shows the results of power analysis based on DPC analysis after Node 1 is omitted. The *x*-axis presents the coupling strength, whereas the *y*-axis presents the percentage of realizations. The analysis is done for a relationship between any two nodes for both directions in the observed five-dimensional sub-network.

**Figure 12:**
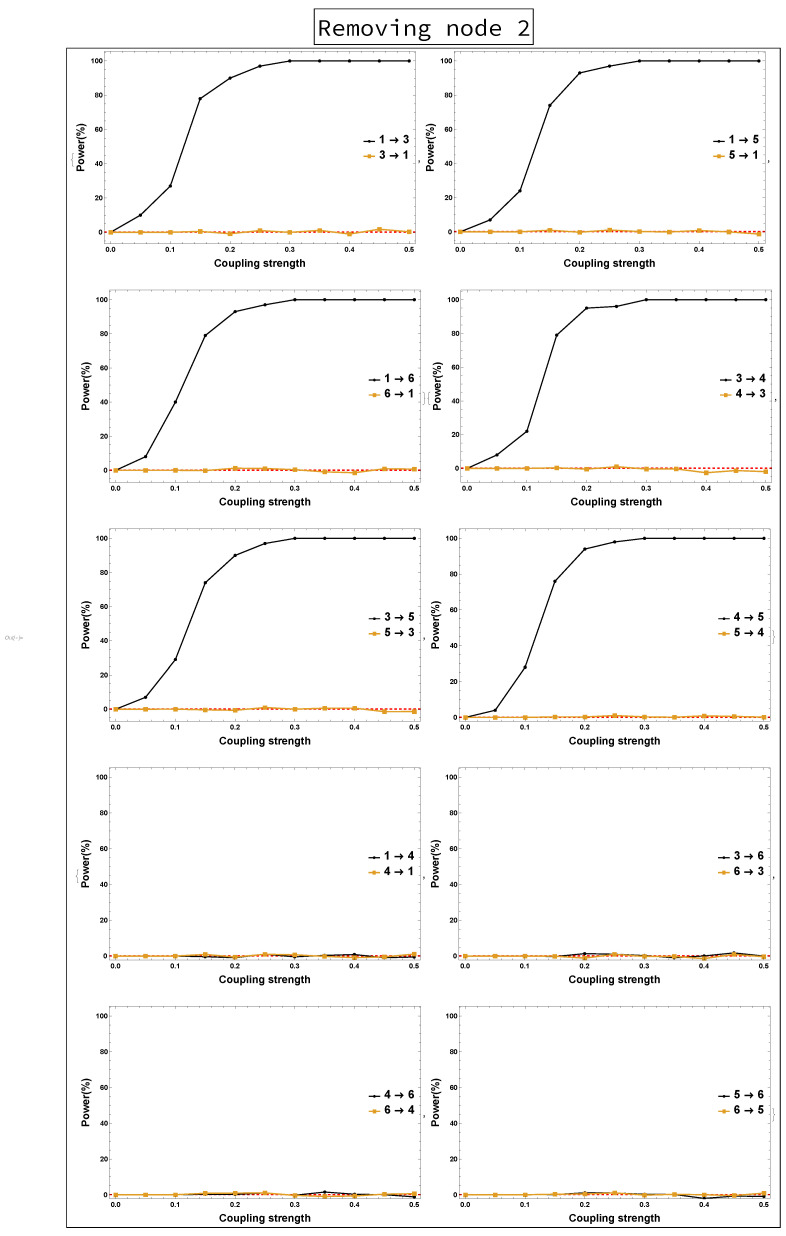
rPDC power curve. The figure shows the results of power analysis based on rPDC analysis after Node 2 is omitted. The *x*-axis presents the coupling strength, whereas the *y*-axis presents the percentage of realizations. The analysis is done for a relationship between any two nodes for both directions in the observed five-dimensional sub-network.

**Figure 13:**
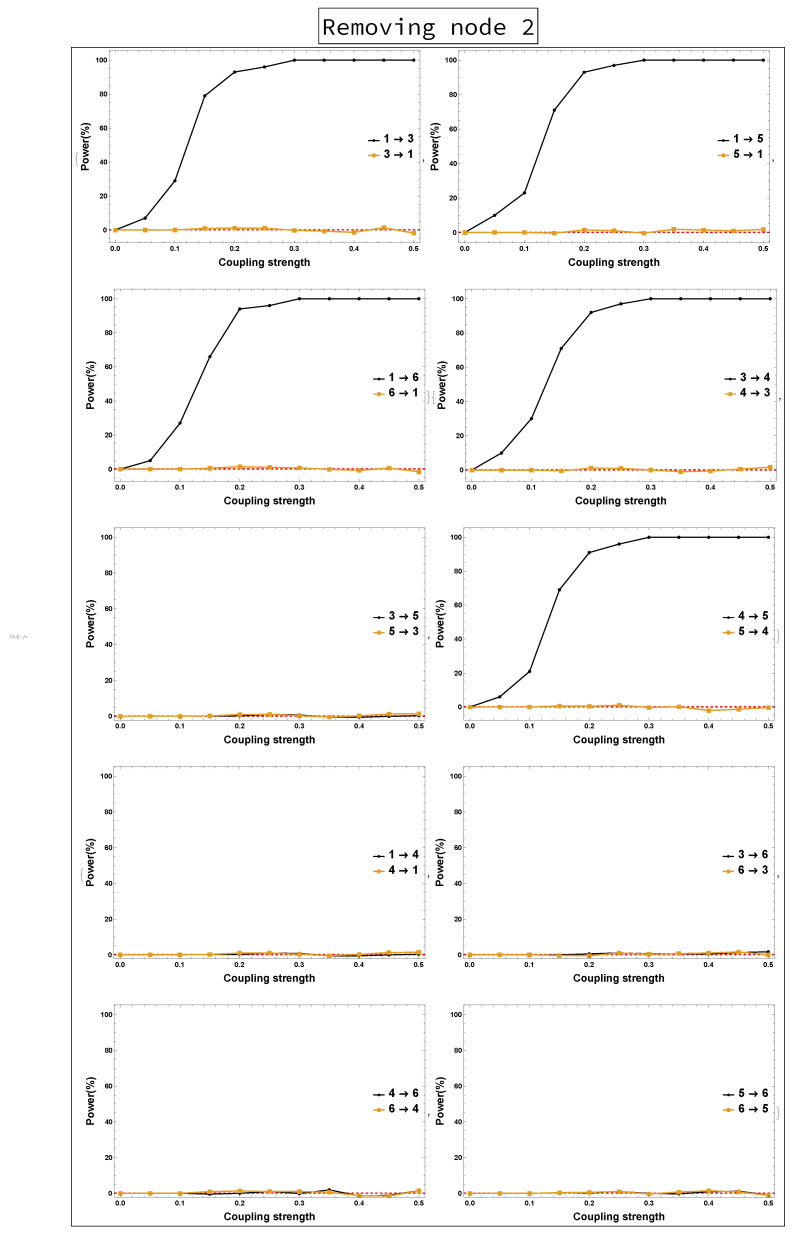
DPC power curve. The figure shows the results of power analysis based on DPC analysis after Node 2 is omitted. The *x*-axis presents the coupling strength, whereas the *y*-axis presents the percentage of realizations. The analysis is done for a relationship between any two nodes for both directions in the observed five-dimensional sub-network.

**Figure 14:**
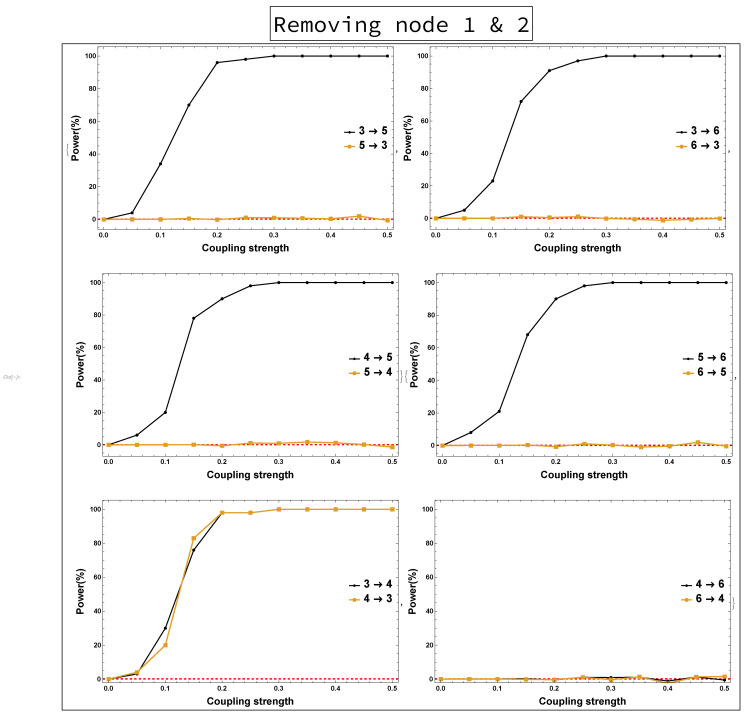
rPDC power curve. The figure shows the results of power analysis based on rPDC analysis after Nodes 1 and 2 are omitted. The *x*-axis presents the coupling strength, whereas the *y*-axis presents the percentage of realizations. The analysis is done for a relationship between any two nodes for both directions in the observed four-dimensional sub-network.

**Figure 15:**
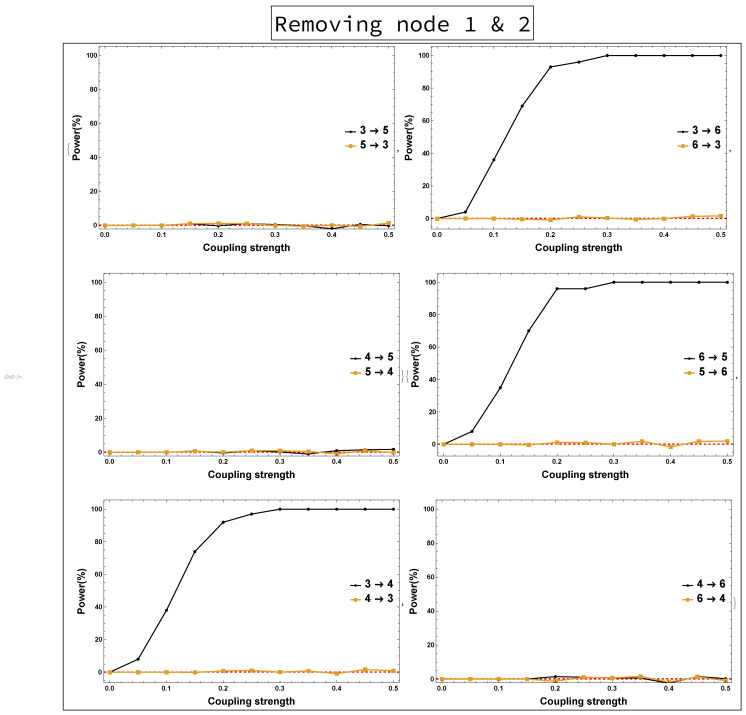
DPC power curve. The figure shows the results of power analysis based on DPC analysis after Nodes 1 and 2 are omitted. The *x*-axis presents the coupling strength, whereas the *y*-axis presents the percentage of realizations. The analysis is done for a relationship between any two nodes for both directions in the observed four-dimensional sub-network.

**Figure 16:**
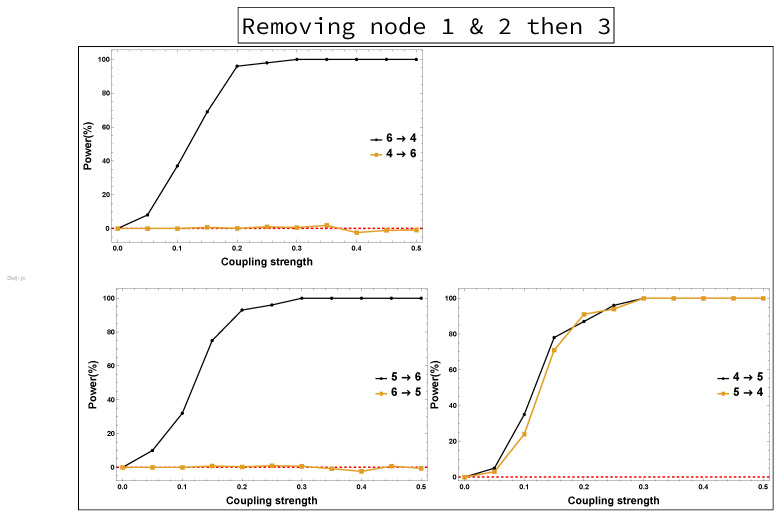
rPDC power curve. The figure shows the results of power analysis based on rPDC analysis after Nodes 1, 2 and 3 are omitted. The *x*-axis presents the coupling strength, whereas the *y*-axis presents the percentage of realizations. The analysis is done for a relationship between any two nodes for both directions in the observed three-dimensional sub-network.

**Figure 17:**
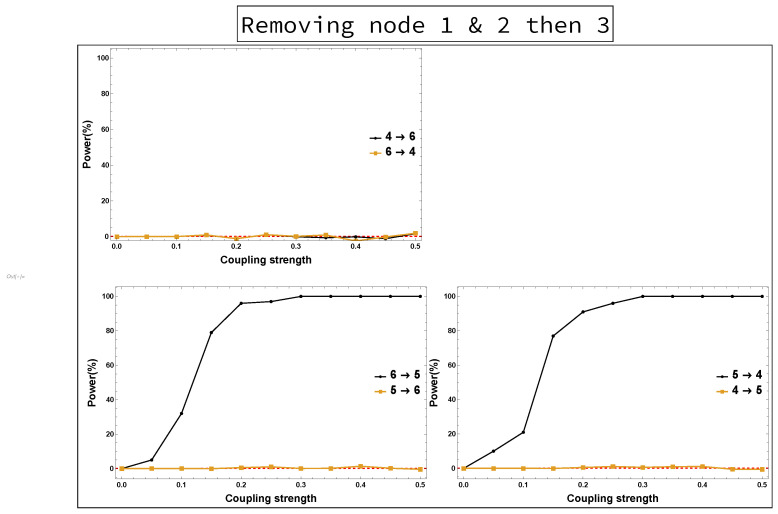
DPC power curve. The figure shows the results of power analysis based on DPC analysis after Nodes 1, 2 and 3 are omitted. The *x*-axis presents the coupling strength, whereas the *y*-axis presents the percentage of realizations. The analysis is done for a relationship between any two nodes for both directions in the observed three-dimensional sub-network.

Power analysis is systematically conducted for both rPDC and DPC for each of the four scenarios for each causal link. The four scenarios are as follows: removing Node 1, removing Node 2, removing Node 1 and Node 2, and removing Nodes 1, 2, and 3. The results of power analysis of the four scenarios are displayed, respectively, in Figure 10, Figure 11, Figure 12, Figure 13, Figure 14, Figure 15, Figure 16 and Figure 17. In each figure, the *x*-axis represents the coupling strength, while the *y*-axis represents the power percentage of realizations. In addition, the red-dashed line displays 5% of the simulated realizations. These results confirm that causal influences are accurately revealed, whereas false positives are controlled.

## 5. Conclusions

The investigation of causal interactions in multivariate systems is of interest for practical applications in many fields. However, including all relevant components of a system is almost impossible in reality. The main problem appears when not observing some components which are important in the underlying system. This, in turn, might lead to misleading conclusions. In networks analysis, the importance of a node depends on the number of links connected to this node. To identify the importance of nodes, the degree centrality measure was utilized. The Out-degree centrality metric was chosen for finding the most influential nodes that correspond to the highest out-degree.

To investigate the implications of having unobserved important nodes, two of the most recent Granger-causality techniques, rPDC and DPC, were employed and compared. Furthermore, the extent to which the existence of important components not included in the analysis, or even if they are unobservable, can be accurately inferred was investigated. The results showed that rPDC outperforms DPC in inferring the existence of unobserved important components. Interestingly, a feedback structure in sub-networks was captured and this, in turn, is considered key to inferring the existence of unobserved important nodes. These results were validated by employing power analysis. This was done to validate every inferred relationship between every two nodes for both directions. The results of Power analysis confirmed that causal influences are accurately revealed, whereas false positives are controlled.

## Data Availability

Not Applicable.
